# Understanding inborn errors of immunity: A lens into the pathophysiology of monogenic inflammatory bowel disease

**DOI:** 10.3389/fimmu.2022.1026511

**Published:** 2022-09-29

**Authors:** Jodie Deborah Ouahed

**Affiliations:** Division of Gastroenterology, Hepatology and Nutrition, Boston Children’s Hospital, Harvard Medical School, Boston, MA, United States

**Keywords:** inborn errors of immunity (IEI), monogenic inflammatory bowel disease, mechanisms of disease, very early onset IBD (VEOIBD), genetics

## Abstract

Inflammatory bowel diseases (IBD) are chronic inflammatory conditions of the gastrointestinal tract, including Crohn’s disease, ulcerative colitis and inflammatory bowel disease-undefined (IBD-U). IBD are understood to be multifactorial, involving genetic, immune, microbial and environmental factors. Advances in next generation sequencing facilitated the growing identification of over 80 monogenic causes of IBD, many of which overlap with Inborn errors of immunity (IEI); Approximately a third of currently identified IEI result in gastrointestinal manifestations, many of which are inflammatory in nature, such as IBD. Indeed, the gastrointestinal tract represents an opportune system to study IEI as it consists of the largest mass of lymphoid tissue in the body and employs a thin layer of intestinal epithelial cells as the critical barrier between the intestinal lumen and the host. In this mini-review, a selection of pertinent IEI resulting in monogenic IBD is described involving disorders in the intestinal epithelial barrier, phagocytosis, T and B cell defects, as well as those impairing central and peripheral tolerance. The contribution of disrupted gut-microbiota-host interactions in disturbing intestinal homeostasis among patients with intestinal disease is also discussed. The molecular mechanisms driving pathogenesis are reviewed along with the personalized therapeutic interventions and investigational avenues this growing knowledge has enabled.

## Introduction

The gastrointestinal tract juggles many roles including absorption of nutrients, transport of electrolytes and fluids, all while maintaining mucosal homeostasis. It is perhaps not surprising that it harbors the largest collection of immune cells in the body, sophistically organized to orchestrate an appropriate immune response against harmful pathogens, while permitting tolerance toward commensal organisms. The immune compartments of the gastrointestinal tract have been categorized into: inductive sites and effector sites (1). *Inductive sites* include the mesenteric lymph nodes and the gut associated lymphoid tissue armored with T cells, B cells and innate immune cells such as mast cells, granulocytes, dendritic cells and group 3 innate lymphoid cells. It is in these inductive sites where adaptive immune cells undergo priming and differentiation. This works alongside *effector sites*, such as the lamina propria and the epithelium, where primed adaptive immune cells reside to assure appropriate immunity against harmful pathogens (1).

Inborn errors of immunity (IEI) are monogenic germline mutations that result in primary immunodeficies (2). Well over 400 IEI have been identified (3), with roughly a third involving a gastrointestinal manifestation, most being inflammatory in nature (4, 5). Inflammatory bowel diseases (IBD) are chronic inflammatory diseases of the gastrointestinal tract. They include Crohn’s disease (CD), a transmural disease, that can affect the mouth to the anus; ulcerative colitis (UC) that results in mucosal inflammation of the colon; and IBD-undefined comprising a mixed picture. The pathophysiology of IBD is believed to involve one’s immune system, genetic predisposition, microbiome and environmental factors.

There are over 80 monogenic causes of IBD (6–8), most of which are IEI (**Table 1**). Consequences of the breakdown of the immune system are robustly reflected in the gastrointestinal tract and often result in disease at an especially young age. Monogenic IBD is predominantly identified in patients diagnosed prior to 6 years old, known as very early onset IBD (VEOIBD) (9). Two large North American centers identified the prevalence of monogenic VEOIBD to be roughly 8% (10, 11), but this varies depending on the population studied (12, 13).

**Table 1:** Monogenic causes of inflammatory bowel disease revealing strong overlap among inborn errors of immunity.

Monogenic cause of IBD	Disorder	Concurrent inborn error of immunity (IEI)
***ADA***	**Atypical combined immune deficiency (CID)**	**Yes**
***ADAM17***	**Inflammatory skin and bowel disease**	**Yes**
***AICDA***	**Immunodeficiency with Hyper-IgM**	**Yes**
***ALPI***	**Intestinal Alkaline Phosphatase deficiency**	**Yes**
***ARPC1B***	**Wiskott-Aldrich syndrome-like**	**Yes**
***BTK***	**Agammaglobulinemia, X-linked 1**	**Yes**
***CASP8***	**Caspase 8 deficiency**	**Yes**
***CD3G***	**Atypical Severe combined immune deficiency (SCID)**	**Yes**
***CD40LG***	**Immunodeficiency, X-linked with hyper-IgM**	**Yes**
***CD55***	**CHAPLE syndrome**	**Yes**
*COL7A1*	Dystrophic epidermolysis bullosa	No
***CTLA4***	**Autoimmune lymphoproliferative syndrome, type V**	**Yes**
***CYBA***	**Chronic granulomatous disease**	**Yes**
***CYBB***	**Chronic granulomatous disease**	**Yes**
***DCLREIC***	**Omenn syndrome (Artemis deficiency)**	**Yes**
***DKC1***	**Dyskeratosis congenita-Hoyeraal Hriedarsson Syndrome**	**Yes**
***DOCK8***	**Dedicator of Cytokinesis 8 (DOCK8) deficiency**	**Yes**
*DUOX2*	DUOX2 deficiency	No
***FCN3***	**Ficolin 3 deficiency**	**Yes**
***FERMT1***	**Kindler syndrome**	**Yes**
***FOXP3***	**Immunodysregulation polyendocrinopathy enteropathy X-linked (IPEX) syndrome**	**Yes**
***G6PC3***	**Congenital neutropenia**	**Yes**
*GUCY2C*	Familial diarrhea	No
*HPS1*	Hermansky-Pudlak syndrome (Type 1)	No
*HPS4*	Hermansky-Pudlak syndrome (Type 4)	No
*HPS6*	Hermansky-Pudlak syndrome (Type 6)	No
***ICOS***	**Inducible co-stimulator (ICOS) deficiency**	**Yes**
***IKBKG***	**X-linked ectodermal dysplasia, anhidrotic and immunodeficiency**	**Yes**
***IL10***	**IL10 deficiency**	**Yes**
***IL10RA***	**IL10 receptor deficiency**	**Yes**
***IL10RB***	**IL10 receptor deficiency**	**Yes**
***IL21***	**IL21 deficiency**	**Yes**
***IL2RB***	**IPEX-like disorder**	**Yes**
***IL2RA***	**IL2RB immune dysregulation**	**Yes**
***IL2RG***	**Atypical SCID**	**Yes**
***ITCH***	**ITCH deficiency**	**Yes**
***ITGB2***	**Leukocyte Adhesion Deficiency I**	**Yes**
***LIG4***	**Atypical SCID**	**Yes**
***LRBA***	**Combined variable immunodeficiency**	**Yes**
***MALT1***	**MALT1 (MALT1 deficiency; IPEX-like)**	**Yes**
***MASP2***	**Mannan Binding Lectin Serine Peptidase 2 defect**	**Yes**
***MEFV***	**Familial Mediterranean Fever**	**Yes**
***MVK***	**Mevalonate Kinase Deficiency**	**Yes**
***NCF1***	**Chronic granulomatous disease**	**Yes**
***NCF2***	**Chronic granulomatous disease**	**Yes**
***NCF4***	**Chronic granulomatous disease**	**Yes**
***NLRC4***	**Autoinflammation with infantile enterocolitis**	**Yes**
*NOX1*	NOX1 deficiency	No
*NPC1*	Niemann-Pick type C	No
***ORAI1***	**ORAI-1 deficiency**	**Yes**
***PIK3CD***	**PIK3CD deficiency and activated PI3K delta syndrome (APDS)**	**Yes**
***PIK3R1***	**Agammaglobulinemia Type 7 and activated PI3K syndrome**	**Yes**
*PLA2G4A*	Cryptogenic multifocal ulcerative stenosis enteritis (CMUSE)	No
***PLCG2***	**Autoinflammation, antibody deficiency and immune dysregulation syndrome**	**Yes**
***POLA1***	**PDR syndrome (pigmentary disorder, reticulate, with systemic manifestation)**	**Yes**
***PTEN***	**PTEN Hamartoma Tumor Syndrome (PHTS)**	**Yes**
***RAG1***	**Atypical SCID**	**Yes**
***RAG2***	**Atypical SCID**	**Yes**
***RIPK1***	**RIPK1 deficiency**	**Yes**
***RTEL1***	**Dyskeratosis congenita-Hoyerall Hriedarsson Syndrome**	**Yes**
***SH2D1A***	**X-linked lymphoproliferative syndrome (XLP1)**	**Yes**
***SKIV2L***	**Trichohepatoenteric Syndrome 2**	**Yes**
*SLC37A4*	Glycogen storage disease type 1b	No
*SLC9A3*	Congenital diarrhea	No
*SLC02A1*	Prostaglandin transporter deficiency	No
***STAT1***	**IPEX-like**	**Yes**
***STAT3***	**Autoimmune disease, multisystem, infantile-onset, 1**	**Yes**
***STIM1***	**STIM1 deficiency**	**Yes**
*STXBP2*	**Familial hemophagocytic lymphohistiocytosis type 5**	**Yes**
*STXBP3*	Syntaxin binding protein 3 defect	No
***TGFB1***	**TGFB1 deficiency**	**Yes**
***TGFBR1***	**Loeys-Dietz syndrome 1**	**Yes**
***TGFBR2***	**Loeys-Dietz syndrome 1**	**Yes**
***TNFAIP3***	**Autoinflammatory syndrome, familial Behcet-like Syndrome**	**Yes**
***TRIM22***	**TRIM22 defect**	**Yes**
***TRNT1***	**sideroblastic anemia, immunodeficiency, periodic fevers, and developmental delay (SIFD)**	**Yes**
***TTC37***	**Trichohepatoenteric syndrome 1**	**Yes**
***TTC7A***	**TTC7A deficiency**	**Yes**
***WAS***	**Wiskott-Aldrich Syndrome**	**Yes**
***XIAP***	**X-linked lymphoproliferative syndrome 2 (SLP2)**	**Yes**
***ZAP70***	**Atypical SCID**	**Yes**
***ZBTB24***	**Immunodeficiency, centromeric instability and facial anomalies (ICF) syndrome**	**Yes**

Monogenic causes of IBD that are concurrent IEI are in bold. IEI are defined as per Tangye et al Human Inborn Errors of Immunity: 2022 Update on the Classification from the International Union of Immunological Societies Expert Committee. J Clin Immunol. 20223. CHAPLE, CD55 deficiency with hyper-activation of complement angiopathic thrombosis, and severe protein-losing enteropathy; IL, interleukin; ITCH, itchy E3 ubiquitin protein ligase; MALT1, mucosa-associated lymphoid tissue lymphoma-translocation gene 1; NOX1, nicotinamide adenine dinucleotide phosphate oxidase 1; PIK3CD, Phosphatidylinositol-4,5-Bisphosphate 3-Kinase Catalytic Subunit Delta; PIK3R1, Phosphoinositide-3-Kinase Regulatory Subunit 1; PI3K phosphoinositide-3-kinase; PTEN, Phosphatase and tensin homolog; RIPK1, receptor interacting serine/threonine kinase 1; STIM1, stromal interaction molecule 1; TGFB1, transforming growth factor Beta 1; TRIM22, Tripartite motif containing 22; TTC7A, tetratricopeptide repeat domain 7A.

Interleukin 10 (IL10) signaling defects exemplify IEI where life-threatening infantile-onset IBD is the most striking feature (14). However, in most cases of IEI resulting in IBD, intestinal inflammation represents one of many disorders. IBD from underlying IEI can be categorized as: epithelial barrier defects that lead to mucosal inflammation; phagocytic defects that lead to alterations in pathogen clearance; T and B cell defects that present with intestinal inflammation; and defects in central and peripheral tolerance that lead to disruption of intestinal homeostasis. Inclusion in one group does not preclude function in another. In this non-exhaustive mini-review, a selection of IEI from each of these categories is described, along with mechanisms driving disease, and associated therapeutic implications (**Table 2**). Importance of the gut microbiota in intestinal inflammation is highlighted.

**Table 2:** Selection of IEI resulting in IBD, histologic features and molecular mechanisms of intestinal disease and summary of available cures and therapeutics warranting further investigation.

Immune category	Immune subcategory	Genes involved	IBD phenotype and other gastrointestinal manifestations	Histologic features of intestinal disease	Molecular mechanism of disease	Established cures for intestinal disease	Therapeutic avenues warranting investigation for managing intestinal disease
**Disordered Epithelial barrier**	Defects in epithelial organization	*FERMT1*	Ulcerative colitis; Strictures of esophagus, ileum and esophagus.	Focal detachment of the epithelium; Altered polarity; Extensive ulceration; Increased mitotic activity.	Dysfunctional kindlin-1 fails to adequately anchor the actin cytoskeleton to the extracellular matrix; Abnormal cell adhesion and polarity.	Nil	Consideration for gene therapy and protein replacement.
	Intrinsic cell defects	*STXBP2; STXBP3*	Life-threatening enteropathy and colitis.	Lymphoplasmacytic inflammation; Neutrophilic crypt abscesses; Villous atrophy; Eosinophilia; Apoptosis; Crypt destruction.	Impaired intracellular vesicular trafficking; Abnormal epithelial cell polarization.	BMT may have a role in some; *STXBP3*: Colectomy is curative in some.	Consideration for gene therapy.
	Defects in epithelial cell death	*TTC7A*	MIA Life-threatening VEOIBD of small and large intestine.	Intestinal atresias: Crypt degeneration; Marked apoptosis; Hypertrophy of the muscularis mucosa; Spindle cell nodules.	Increased susceptibility to apoptosis and aberrant intestinal epithelial polarity secondary to impaired localization of PI4KIIIα to the plasma membrane and aberrant AKT signaling.	Nil	Leflunamide; Rho kinase inhibitors; AKT signaling agonists.
**Defects in phagocytosis**		*CYBA*, *CYBB*, CYBC1, *NCF1*, *NCF2*, *NCF4*	Inflammatory and fistulizing Crohn’s disease; Strictures and obstructions of the esophagus and pylorus.	Microgranulomas; Pigmented macrophages; Eosinophilia; Villous shortening.	Impaired phagocytosis; Reduced memory B cells; Defective autophagy; Increased inflammasome activation.	BMT	Anti-IL1 therapy; Gene therapy; IFNγ therapy.
**Defect in T and B cells**		*DKC1* and *RTEL1*	Colitis Enteropathy Strictures of the esophagus and rectum.	Increased apoptosis.	Bone marrow failure; Intrinsic epithelial cell defects.	Nil	Wnt agonists.
		*RAG1* *RAG2*	Infantile-onset colitis.	Not well characterized in literature.	Proliferation of T lymphocytes with impaired adaptive immunity; Impaired central tolerance; Defective generation of Treg; Allergic inflammation.	BMT	Gene therapy and gene editing.
**Disorders affecting central and peripheral tolerance**	Disorders of central tolerance	*AIRE*	No IBD. Features of intestinal dysfunction (diarrhea, constipation, enteropathy, malabsorption).	Absence of enteroendocrine cells.	Impaired central tolerance resulting in autoreactive T cells and autoimmunity.	Nil	Gene editing of iPSCs.
	Disorders of peripheral tolerance	*FOXP3*	Early onset enteropathy and occasionally colitis.	Severe villous atrophy; Extensive eosinophilic and lymphocytic infiltrate.	Impaired Treg function.	BMT	Lentiviral *FOXP3* gene transfer of autologous CD4^+^T cells.

BMT, bone marrow transplant; IBD, inflammatory bowel disease; iPSC, induced pluripotent stem cells; MIA, multiple intestinal atresias; PI4KIIIα, phosphatidylinositol 4 phosphatdylinositol 4-kinase III alpha; Treg, T regulatory cell; VEOBID very early onset inflammatory bowel disease.

## Disordered epithelial barrier

The intestinal epithelial barrier consists of a single layer of epithelial cells, a mucous layer, and an organization of immune cells. A delicate balance of cell turnover is imperative for appropriate barrier function. Disruption of the epithelial barrier can be subdivided into: defects in epithelial organization; intrinsic cell defects; and defects in epithelial cell death (8).

### Defects in epithelial organization

Mutations in *KINDLIN-1*, also known as *FERMT1*, result in kindler syndrome, an IEI that manifests primarily as a skin disorder, characterized by acral blistering as neonates and poikiloderma with age (15). Gastrointestinal manifestations include anal, esophageal and ileal strictures (16, 17). Approximately 15% of patients develop UC (18). Kindlin-1 knock out mice exhibit severe colitis with extensive epithelial detachment, ensuing perinatal lethality (19). In humans, the severity of UC is milder and does not correlate with specific genotypes (20).

The molecular basis of kindler syndrome results from dysfunctional kindlin-1, a cytoplasmic adaptor protein that normally anchors the actin cytoskeleton to the extracellular matrix (15, 21, 22). It is important in cell adhesion and polarity (23). In the colon and rectum, kindlin-1 is localized in the periphery of epithelial cells, while there is only minimal expression in the terminal ileum (23), consistent with patients’ colitis phenotype. Microscopically, the colon displays extensive ulceration with focal detachment and loss of epithelium from the underlying tissue, along with altered cell polarity (23), increased mitotic activity and infiltration of plasma cells and eosinophils (23, 24). The breach of intestinal barrier is thought to result in penetration of antigens, inflammation, and mucosal changes (23).

While the contribution of barrier dysfunction to mechanisms generating IBD remains incompletely understood, mutations in *FERMT1* illustrate how breakdown of this barrier can result in colitis (23). At present there is no cure for kindler syndrome. In some, colitis is managed with anti-inflammatory agents (16), others respond to immune modulators (25), while others require colectomy (23). Potential therapeutic avenues include gene therapy or protein replacement (26).

### Intrinsic cell defects

Mutations in *STXBP2* result in an IEI responsible for familial hemophagocytic lymphohistiocytosis, often associated with hypogammaglobulinemia, hearing loss, and bleeding tendencies (27, 28). About 38% of patients develop IBD, characterized by enteropathy and colitis, often within months of life (28, 29). There is no identifiable genotype-phenotype correlation (30). STXBP2 is expressed in intestinal epithelial cells (IECs) and is important for regulating intracellular granule trafficking and docking at the plasma membrane (29, 31, 32). Damaging mutations in *STXBP2* result in impaired degranulation of NK cells and cytotoxic T cells, ensuing compromised cytotoxicity (33, 34) and diminished granule fusion in neutrophils, resulting in impaired bacterial killing (35). Additionally, mutations in *STXBP2* result in altered epithelial cell polarity (29, 30).

Damaging mutations in *STXBP3*, a related gene also important in regulating intracellular vesicular trafficking, results in life-threatening infantile-onset IBD of both large and small intestine, usually associated with hearing loss (36). STXBP3 is expressed in epithelial and immune cells and is required for IEC polarization (36). Intestinal disease is histologically characterized by lymphoplasmacytic inflammation, neutrophilic crypt abscesses, villous atrophy, eosinophilia, apoptosis, and crypt destruction (36). Colitis is refractory to immune suppression (36). One patient described exhibited remission of colitis following bone marrow transplant (BMT), while colectomies were curative in four (36).

The unifying molecular functions of STXBP2 and STXBP3 point to their importance in membrane trafficking and maintaining a polarized IEC layer. Disruption in polarity seems, at least partly, responsible for the IBD that persists in a subset of patients with mutations in *STXBP2* following successful BMT (29). Additional studies are necessary to delineate mechanisms and cellular components whereby STXBP2 and STXBP3 maintain mucosal homeostasis.

### Defects in epithelial cell death

Patients with damaging mutations in *TTC7A* can present with multiple intestinal atresias (MIA) of the large and small intestine (37), VEOIBD, and a combined immune deficiency (CID) (38–41). VEOIBD from TTC7A deficiency results in profuse bloody diarrhea shortly after birth, usually necessitating parenteral nutrition (39–41). Intestinal disease is microscopically characterized by atresias, architectural distortion, crypt degeneration, pronounced apoptosis, hypertrophy of the muscularis mucosa and spindle cell nodules (42). While all patients present with intestinal manifestations (MIA or VEOIBD, with or without CID), it remains unclear what drives the phenotypic variability.

TTC7A is a scaffolding protein normally expressed in the plasma membrane of intestinal cells (40). It localizes phosphatidylinositol 4 phosphatdylinositol 4-kinase III alpha (PI4KIIIα) to the plasma membrane to facilitate synthesis of PI4-phosphate (40). PI4-phosphate is an upstream precursor of the AKT signaling pathway, critical in preventing apoptosis and promoting proliferation (43). TTC7A deficiency thereby results in increased susceptibility to apoptosis, consistent with patients’ histologic features (40), and aberrant IEC polarity, in line with intestinal atresias (38). Intestinal organoids derived from patients demonstrate disrupted apico-basal polarity, poor epithelial cell integrity, reduced proliferation and absence of luminal space (38). This illustrates the damaging consequences of deficient TTC7A on the intestinal epithelial barrier, in absence of effects from the microbiome and immune system. TTC7A is also expressed in the thymus, but its contribution to CID remains undefined (44).

TTC7A deficiency is fatal in over 50% of patients (45). While BMT can correct the CID, it does not rectify intestinal disease (39, 41, 45), which remains life-threatening (38, 40, 41, 46). VEOIBD is resistant to immune suppression (41, 45) and MIA recur following surgical resection (47). Administration of a RhoA kinase inhibitor to patient-derived organoids reverses defects in polarity and ameliorates proliferation *via* mechanisms that remain to be elucidated (38). Jardine et al. identified that leflunomide reduces apoptosis, optimizes structure and ion transport in patient-derived colonoids and also restores gut function in a *ttc7a^-/-^* zebrafish model (48). While the mechanism remains unclear, leflunomide rescues defective AKT signaling, critical in preventing apoptosis and promoting proliferation (43, 48). TTC7A deficiency highlights the importance of these functions in maintaining mucosal homeostasis.

## Disordered phagocytic functions

The nicotinamide adenine dinucleotide phosphate (NADPH) oxidase complex, normally generates hydrogen peroxide and reactive oxygen species in phagocytes. Damaging variants in genes encoding components of the NADPH oxidase complex (*CYBA*, *CYBB*, CYBC1 (49), *NCF1*, *NCF2*, and *NCF4)* result in impaired anti-microbial activities in phagocytes (50), and thereby chronic granulomatous disease (CGD) (51). Patients have increased susceptibility to infections, especially catalase positive bacteria and fungi (52). Intestinal manifestations include inflammatory and fistulizing CD, which can be mistaken for non-monogenic IBD (51, 53–55). The colon is usually affected, histologically characterized by microgranulomas, pigmented macrophages, eosinophilia and occasionally the small intestine displays villous shortening (56). Esophageal, pyloric and intestinal strictures and obstructions are additional gastrointestinal manifestations (56–59).

The specific mechanisms driving intestinal inflammation in patients with CGD remains to be elucidated. Patient survival is related to the amount of residual NADPH oxidase function (60). In addition to impaired phagocytosis, evidence has pointed to reduced memory B cells (61), defective autophagy and increased inflammasome activation with increased IL1β activity (62, 63).

BMT is curative for both susceptibility to infections and IBD (64–66). Yet, prior to transplant, management of CD in CGD patients is challenging. One needs to balance the inherent high susceptibility to infection with need for immune suppression to control intestinal inflammation. Anti-TNF agents, conventionally used in CD, are avoided given risk of life-threatening infection (67). To minimize extra-intestinal immune suppression, the gut-specific agent, Vedolizumab (an anti-α4β7 integrin blocker), has been tried, but does not result in endoscopic improvement (68). Anti-IL1 agents have been used in small numbers, and while it restores autophagy, reports on whether it improves colitis are mixed (62, 63, 69). This is being further studied (70). IFNγ is used in CGD patients for infection prophylaxis by stimulating superoxide release, but its effects on IBD remains unclear (71, 72). Gene therapy is being investigated (73).

## Defects in T and B cells

Mutations in *DKC1* and *RTEL1*, result in dyskeratosis congenita (DC), telomeropathies with accelerated shortening or damage of telomeres and bone marrow failure (74–76). The classic clinical triad is dysplastic nails, abnormal skin pigmentation and oral leukoplakia (74). Other features include pulmonary fibrosis, liver disease, and cancer predisposition (74, 77, 78). Hoyeraal-Hreidarsson syndrome is a severe form of DC with intrauterine growth retardation, microcephaly, cerebellar hypoplasia and often VEOIBD (78–81), in addition to esophageal and rectal strictures (79–81). Patients with DC have progressive T, B and NK cell lymphopenia (77–81). Mutations in *DKC1* result in medically refractory enterocolitis (76, 80, 81). Similarly, mutated *RTEL1* leads to refractory colitis or enteropathy (74, 75, 77). Histologically, intestinal disease is characterized by increased apoptosis, whether attributed to mutations in *DKC1* (76, 80) or *RTEL1* (74, 77).

The molecular basis of IBD in DC is incompletely understood. DKC1 encodes dyskerin, which is ubiquitously expressed, including the colon, small intestine and peripheral blood cells (82). Dyskerin is important in ribosome biogenesis, preserving telomere integrity (83), responding to DNA damage (84), and vesicular trafficking (85). RTEL1 is expressed in murine intestinal crypts, where intestinal stem cells reside, which are imperative in intestinal homeostasis given their self-renewal capacity (86). RTEL1 is essential for DNA replication and telomere maintenance (87). RTEL1-deficient cells exhibit spontaneous apoptosis and senescence (77).

The course of IBD following BMT in DC patients is poorly described: some report resolution in patients with *RTEL1* mutation (77), while IBD persisted in at least one patient with *DKC1* mutation (81 *).* This variable response suggests that the underlying mechanism of disease may extend beyond effects of bone marrow failure. This notion is supported by identifying that intestinal organoids generated from a patient with mutated *DKC1* exhibit poor growth and differentiation with decreased epithelial markers and reduced Wnt signaling, resulting in attenuated intestinal stem cell renewal (88). These features, observable in absence of input of the immune system, reversed following treatment with a Wnt agonist (88). Similar reversible features were recapitulated in mice lacking telomerase when treated with a Wnt agonist (89). This suggests that telomeropathies have intrinsic epithelial defects in addition to immune deficiencies and bone marrow failure that contribute to IBD. These findings can be harnessed by considering Wnt agonists in the management of this medically refractory IBD (90, 91).

Omenn syndrome (OS), is an IEI that typically presents within weeks of life with immunodeficiency, lymphadenopathy, hepatosplenomegaly, erythroderma and fever, often with infantile-onset colitis (92–95). Patients have a dysplastic thymus, impaired central tolerance resulting in oligoclonality of T cells (2, 95) and absent mature T and B cells (96, 97). OS can result from various IEI, including mutations in *RAG1* and *RAG2* responsible for somatic V(D)J recombination which defines B and T cell repertoire (95). *RAG1* and *RAG2* are primarily expressed in T and B lymphocytes (98–100). Genotype-phenotype correlations are variable (101).

The mechanisms driving IBD in OS are unclear. It is thought that impaired central tolerance, proliferation of T lymphocytes with impaired adaptive immunity, defective generation of immune suppressing T regulatory cells (Treg), absent mature T and B cells, and allergic inflammation are all involved (95). Murine models of OS exhibit prominent gastrointestinal inflammation driven by T cells, as their adoptive transfer in immunodeficient hosts suffices to result in colitis, whereas depletion of CD4^+^ T cells improves their intestinal inflammation (102).

BMT reverses intestinal inflammation and cures OS, which is otherwise fatal (103). The striking life-threatening disease in OS illustrates the critical role of mature T and B cells in the gastrointestinal tract and beyond. Gene therapy and gene editing are potential therapeutic approaches (104).

## Disorders affecting central and peripheral tolerance

### Disorders of central tolerance

While OS holds features of disordered central tolerance, this is better illustrated by mutations in *AIRE* resulting in autoimmune polyendocrinopathy, candidiasis ecto-dermal dystrophy (APECED). AIRE is normally expressed in the medulla of the thymus and drives negative selection of autoreactive T cells (105). Patients manifest autoimmunity of endocrine and non-endocrine tissues and chronic mucocutaneous candidiasis (106). While patients do not develop IBD, they experience various gastrointestinal disorders: autoimmune hepatitis, atrophic gastritis, malabsorption, and poorly described chronic diarrhea and constipation (106–108). Some exhibit enteropathy with antibodies against tryptophan hydroxylase (109, 110). A unique histologic feature among some APECED patients is loss of enteroendocrine cells (110–112). Studies are warranted to better characterize these gastrointestinal disorders. Symptomatic management is employed as there is no cure. A potential future curative intervention is to restore AIRE expression in patient-derived induced pluripotent stem cells (113).

### Disorders of peripheral tolerance

Aside from central tolerance, peripheral tolerance is also critical for maintaining intestinal homeostasis. Tregs normally maintain peripheral immune tolerance and suppress excessive immune responses (114–118). FOXP3 is critical for the development and function of Tregs (116, 119, 120). FOXP3^+^ cells are densely expressed in lymphoid follicles and scattered in the lamina propria (121). They expand following intestinal inflammation from IBD (121, 122). Immune dysregulation, polyendocrinopathy, enteropathy, X-linked (IPEX) syndrome results from damaging mutations in *FOXP3 (* 123–125). The typical clinical triad is endocrinopathy, enteropathy, and dermatitis, with enteropathy often being the first and most severe phenotype. Intestinal disease most commonly affects the small bowel, but patients can also have colitis (126). The histologic hallmark is severe villous atrophy with extensive eosinophil and lymphocytic infiltrates (126, 127). Other features of IPEX syndrome include: allergies, autoimmune hematologic disorders, hepatitis, nephropathy, and susceptibility to infection (126, 128). There is extensive phenotypic variability, even among patients with identical genotype (126). IPEX syndrome and other IPEX-like diseases (129) underscore the imperative role of peripheral tolerance in maintaining homeostasis in various systems, especially the intestine.

IPEX syndrome can be managed with immune suppression, ideally using sirolimus to spare Tregs (130). However, disease-free survival is poor following immune suppression (126). BMT is currently the only effective cure, with the most significant predictor of survival being the lowest organ involvement (126). A clinical trial is ongoing with lentiviral *FOXP3* gene transfer of autologous CD4^+^T cells (131, 132), hypothesized to reverse the life-threatening multisystemic effects of IPEX syndrome.

## Disrupted gut-microbiota-host interactions

The gut microbiota and host immune system are closely intertwined such that each affects development and function of the other (133–135). The bidirectional crosstalk between the gut microbiota and host immune system is disrupted in IBD. Patients with non-monogenic IBD have dysbiosis (136–138) and reduced microbial diversity (139, 140) which may affect intestinal permeability and promote inflammation (141). Those with an IEI driving IBD also have distinct variations of their microbiome as described in CGD and TTC7A deficiency (142) among others (141). Moreover, microbiota changes have been identified following therapeutic interventions, such as increased bacterial diversity in an IPEX patient after fecal microbial transplantation (FMT) (143) and changes in the microbiome of patients with severe combined immune deficiency after BMT (144). Despite limited efficacy to date, eagerness remains in therapeutically targeting dysbiosis *via* antibiotics, prebiotics, probiotics and FMT in IBD (145–147).

## Conclusion

Herein, a selection of IEI is presented, highlighting how interruption of various components of immunity contribute to IBD development. Detrimental consequences ensue when IEI disrupt the necessary mechanisms that maintain intestinal homeostasis.

Among patients with monogenic IBD, employing conventional therapeutics is oftentimes inadequate. Indeed, biologics are only effective in 25.5% of patients with monogenic IBD (9). Moreover, among VEOIBD patients, monogenic disease is a driver of disease severity, including death (10). The growing number of IEI manifesting with IBD, and interaction with the microbiome permits a deeper understanding of the molecular mechanisms driving intestinal inflammation. This knowledge holds important prospects to facilitate personalized therapeutic options and optimized prognostics (148, 149). In some scenarios, understanding the mechanisms driving intestinal inflammation reveals pathways of importance that merit evaluation for therapeutic targeting. Examples include consideration of leflunomide to rescue defective AKT signaling in patients with TTC7A deficiency and refurbishing IL1 antagonists after learning that CGD involves defective autophagy with increased IL1β activity. In other situations, gained insight serves to avoid potentially life-threatening interventions, such as use of anti-TNF agents in CGD patients, or BMT for patients with epithelial barrier defects. Finally, in other circumstances, curative interventions are enabled in otherwise life-threatening disease, such as BMT for IL10 signaling defects, CGD, IPEX syndrome and OS, and sets the groundwork for investigating gene therapy and manipulation of the microbiome.

Clinicians managing patients with IBD from underlying IEI are challenged to think outside the box, delve into the pathologic mechanism at play, and consider innovative personalized approaches. While understanding the molecular mechanisms of IEI driving IBD inspired critical advancements in personalized therapeutics, there remains an urgent need to further advance this field. Developments have been achieved by studying murine models, intestinal organoids, and transcriptomics. More is attainable by harnessing multi-omic efforts in a collaborative and interdisciplinary fashion.

## Author contributions

The author confirms being the sole contributor of this work and has approved it for publication.

## Funding

Research reported in this publication was supported by the National Institute Of Diabetes And Digestive And Kidney Diseases of the National Institutes of Health under Award Number K08DK122133. The content is solely the responsibility of the authors and does not necessarily represent the official views of the National Institutes of Health.

## Conflict of interest

The author declares that the research was conducted in the absence of any commercial or financial relationships that could be construed as a potential conflict of interest.

## Publisher’s note

All claims expressed in this article are solely those of the authors and do not necessarily represent those of their affiliated organizations, or those of the publisher, the editors and the reviewers. Any product that may be evaluated in this article, or claim that may be made by its manufacturer, is not guaranteed or endorsed by the publisher.
